# Unrevealed Depression Involves Dysfunctional Coping Strategies in Crohn's Disease Patients in Clinical Remission

**DOI:** 10.1155/2016/7803262

**Published:** 2015-12-28

**Authors:** Caterina Viganò, Roberta Calzolari, Paola Marianna Marinaccio, Cristina Bezzio, Federica Furfaro, Gabriella Ba, Giovanni Maconi

**Affiliations:** ^1^Psychiatry Unit, Department of Biomedical and Clinical Sciences, L. Sacco University Hospital, Via G.B. Grassi 74, 20157 Milan, Italy; ^2^Gastroenterology Unit, Department of Biomedical and Clinical Sciences, L. Sacco University Hospital, Via G.B. Grassi 74, 20157 Milan, Italy

## Abstract

*Background and Aims*. This study investigated the proportion of CD patients in clinical remission with clinical depression, and coping strategies in those with severe depressive disorders.* Materials and Methods*. One hundred consecutive CD patients in clinical remission were screened for anxiety and depression by using Hospital Anxiety and Depression Scale and patients with depressive symptoms were further investigated by means of Cognitive Behavioural Assessment 2.0 and Beck Depression Inventory (BDI). Afterwards the coping strategies were assessed through the Brief-COPE questionnaire.* Results*. Twenty-one patients had anxious symptoms and 16 had depressive symptoms with or without anxiety. Seven of these patients (43.8%) showed significant depressive symptoms. Compared to patients without psychiatric disorders, these patients showed significant lower score in “positive reframing” (*p*: 0.017) and in “planning” (*p*: 0.046) and higher score in “use of instrumental social support” (*p* < 0.001), in “denial” scale (*p*: 0.001), and in “use of emotional social support” (*p*: 0.003).* Conclusions*. Depressed CD patients in clinical remission may have dysfunctional coping strategies, meaning that they may not be able to implement functional strategies to manage at best stress related with their disease.

## 1. Introduction

Crohn's disease (CD) is an inflammatory chronic disease that may affect any part of the gastrointestinal tract. Its prevalence is increasing around the world, with an annual incidence ranging from 12.7 to 20.2 per 100,000 person-years, respectively, in Europe and North America, and a prevalence ranging from 319 to 322 per 100,000 persons [1]. Crohn's disease has a variable clinical evolution, characterized by extraintestinal manifestations and intestinal and perianal complications frequently requiring surgical treatment with significant negative impact on quality of life.

A significant proportion of CD patients shows psychiatric comorbidity, in particular depressive and anxious disorders [2, 3]. It has been estimated that the long-life prevalence of the major depression and anxiety disorders can affect more than 30% of patients in clinical remission and up to 60–80% of patients with active CD [2, 4–7]. Furthermore, the role of the depression on the severe impairment of quality of life [7, 8] and in reactivation of the CD [9–11] has been highlighted.

Another important psychosocial factor affecting quality of life and disease reactivation in CD is coping style [12–14]. Coping style is commonly defined as someone's preferred way of dealing with stressful situations. Maladaptive coping is a technique that may reduce symptoms while maintaining and strengthening the disorder. This has been interpreted as an emotional reaction against the pain in terms of dramatization [15] or as a feeling of helplessness to deal with the stressor and relying on others to resolve the stressful event or situation and interpreted as a psychological factor more linked to the emotional sphere, or social habits that can affect the patient's behaviour [16].

A link between depression and dysfunctional coping strategies is well known [17, 18] and it has been reported in several conditions such as rheumatoid arthritis [19], fibromyalgia [20, 21], chronic and arthritis pain [22, 23], cancer [24], HIV patients [25], and patients in the chronic phase after stroke [26], and women undergoing in vitro fertilization [27]. However, their occurrence in CD remains largely uninvestigated.

Therefore, we assessed the prevalence altered coping strategies in a series of hospital-based out-patients with CD in clinical remission with confirmed depressive disorder, in order to identify potential areas of intervention.

## 2. Materials and Methods

### 2.1. Study Design

This prospective study included a series of consecutive patients with endoscopic and histological diagnosis of CD, defined according to the criteria of the literature [28]. These patients were recruited between March and September 2014, during an ambulatory visit at the IBD unit of the Luigi Sacco University Hospital in Milan, a tertiary referral centre for IBD care.

The eligible patients were first screened to identify the presence of anxious/depressive symptoms (Step 1); then patients with depressive symptoms were further investigated to confirm and assess the severity of depression (Step 2). Afterwards the coping strategies of these patients were analysed (Step 3) and compared with those of CD patients without psychiatric symptoms.

### 2.2. Patients

An unselected series of patients aged between 18 and 85 years with confirmed diagnosis of CD, in a status of clinical remission, regularly followed up in our unit, were considered eligible for this study. The patients were excluded if they showed the following conditions: clinically active disease (Crohn's Disease Activity Index (CDAI) > 150), diagnosis of psychiatric disorder preceding the diagnosis of Crohn's disease, pregnant patients, patients with history of substance abuse, or significant comorbidity including the diagnosis of neoplastic diseases.

All the patients included were informed about the nature of the study and gave their consent to process their data. The study was approved by the Ethical Committee and the Review Board of our Hospital.

### 2.3. Evaluation Steps

Eligible patients underwent the following investigations.

**Step 1 .**

The presence of psychological distress, in particular anxiety and/or depressive symptoms, was initially investigated through the Hospital Anxiety and Depression Scale (HADS). The scale is composed of 14 items, 7 for evaluating the anxious symptomatology and 7 for evaluating the depressive symptomatology. Every item can show a score ranging from 0 to 3, resulting in maximum 21 points for both subscale “anxiety” and subscale “depression.” Scores equal to or higher than 11 on each scale were considered indicative of the presence of a disorder; scores from 8 to 10 were considered indicative of a mild symptomatology, or borderline cases, while scores from 0 to 7 were considered indicating a patient without disorders. A meta-analysis of 2010 indicated the values of sensibility and specificity of the HADS in order to identify a Major Depressive Disorder: correspondingly 0.82 and 0.74, using as cut-off the score 8, and 0.56 and 0.92, using as cut-off the score 11. The same meta-analysis found values of sensibility and specificity of 0.78 and 0.74 (with a cut-off set at 8) in order to identify Generalized Anxiety Disorder [29].

**Step 2 .**

The patients with depression with or without anxiety, namely, patients with HADS scores ≥ 8 in the subscale “depression,” were investigated by means of the “questionnaire D” that represents the sheet 8 of the Cognitive Behavioural Assessment (CBA) 2.0. This questionnaire D has been used as further primary screening instrument. The CBA 2.0 is a set of ten sheets investigating some primary psychological constructs such as state anxiety, depression, fears, obsessions, compulsions, and psychophysiological disorders and tends to identify potential dysfunctional areas [30]. The items of each sheet are homogeneous for formal aspect or historical derivation and probe a specific aspect of the patient. The questionnaire D (sheet 8) assesses the depressive symptoms through 24 dichotomous response items (yes/no), among them there are critical items aiming at investigating specifically depressive manifestations of particular clinical interest. The scores higher than 95th percentile are deemed significant. The questionnaire D has a high internal consistency (Cronbach's alpha = 0.87). The constant follow-up of regulatory data and the particular attention paid in building it make the CBA 2.0 one of the best “self-report” instruments for the psychodiagnostic testing (Cronbach's alpha between 0.74 and 0.85 in community and between 0.68 and 0.97 in clinical environment) [31].

In patients that achieved scores higher than the 95th percentile at this, the severity of depression was assessed through the Beck Depression Inventory (BDI). BDI allows investigating in a more detailed and deeper way the patients' depressive symptoms and assesses their severity. The scale is focused on 21 symptomatic areas and the patient is asked about how they have been feeling in the last week. The first 13 items represent the cognitive-affective subscale and the following 13 the subscale of somatic and performance symptoms. Each symptom changes according to four levels (from zero to three) ranging in intensity. The scores allow identifying five descriptions: (a) possible denial of depression, pretending to be well (less than four points); (b) minimal depression (5–9 points); (c) from mild to moderate depression (10–18 points); (d) from moderate to severe depression (19–29 points); (e) severe depression (30–63 points). BDI has an internal consistency between 0.79 and 0.90 depending on the group of patients. Since a score beyond 40 is too high even for a case of severe depression and suggests a possible exaggeration of depression, patients with this score were excluded [32].

Summarizing, Step 1 was to screen the anxiety and depressive symptomatology; that does not mean a psychiatric disorder, but only psychological distress. In the Step 2, the patients with HADS score positive for depression were further investigated to confirm and assess presence and severity of depressive disorder (first the CBA-D test and second Beck Depression Inventory), which scored the severity of cluster symptoms usually used as diagnostic criteria (DSM).

**Step 3 .**

Finally, the coping strategies of the patients who resulted positively after the questionnaire D of the CBA 2.0 and BDI were assessed through the Brief-COPE. The patients' coping style, or rather how they usually cope with the stressing situations, was assessed through the Brief-COPE, an abbreviated version of COPE created by the same author [33]. The Brief-COPE comprises 28 items articulated in 14 scales composed of 2 items each. The items are assessed according to a four-point scale from 1 (I usually do not do this at all) to 4 (I usually do this a lot) and the patient is asked to specify how many times it takes to implement the proposed behaviour before a stressing situation. Compared to the extended version (COPE), the Brief-COPE does not comprise the less important items in the factor analysis and/or those resulting in being less clear or hard to be understood; furthermore a “self-blame” scale was added that had resulted in being important from previous experiences. The psychometric characteristics of the scales composing of the Brief-COPE resulted in being satisfactory and also the validity was provided with documentary evidence [33].

### 2.4. Statistical Analysis

The quantitative data were presented as median and interquartile range (or IQR that is a measure of statistical dispersion, being equal to the difference between the upper and the lower quartiles, IQR = Q_75_  − Q_25_). Comparison of scores between groups has been performed using Mann-Whitney *U* test for nonparametric data.

A value of *p* < 0.05 was deemed as statistically significant.

## 3. Results

### 3.1. Patients' Characteristics

Out of 100 patients initially included in the study, 5 were excluded because of a diagnosis psychiatric disorder before the diagnosis of CD. Demographic and clinical features of the 95 patients included in the study are shown in Table 1.

### 3.2. Prevalence of Psychiatric Disorders

**Figure 1:**
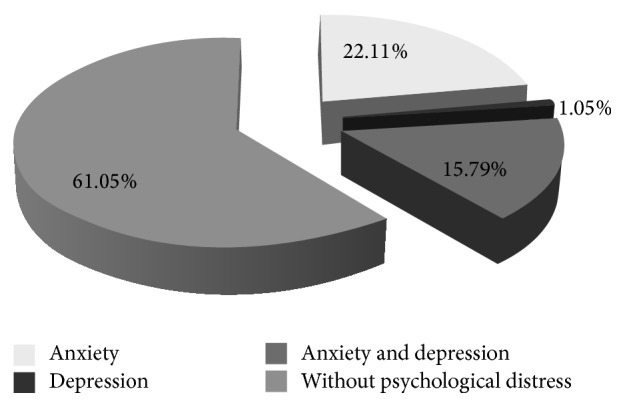
Prevalence of anxiety or depression in the population included in the study.

Thirty-seven patients showed depressive or anxiety symptoms (HADS score on one of both subscales ≥ 8). In particular, 21 patients (22.1%) showed anxiety, 1 patient showed depressive symptoms, and 15 patients (15.79%) showed both conditions (Figure 1).

Six patients (6.3%) have been already treated for anxiety or depressive symptoms: one patient with benzodiazepine, 1 patients with antidepressant SSRI, and 2 patients with a combination of antidepressants (SSRI) and benzodiazepine and 2 did not remember the treatment. Three of these patients showed a residual symptomatology in the Hospital Anxiety and Depression Scale (scores ≥ 8).

### 3.3. Severity of Depressive Symptoms

The 16 patients with scores higher than or equal to eight in the subscale “depression” HADS were investigated with the questionnaire D (QD) of the Cognitive Behavioural Assessment 2.0 (sheet 8) and 7 (43.8%) showed relevant depressive symptoms (scores > 95th percentile in the QD); none of these patients has ever been treated for depressive disorders.

These patients were submitted to the Beck Depression Inventory that confirmed a severe depression (BDI score between 30 and 40 points) in 2 patients, a moderate-severe depression (BDI score between 19 and 29 points) in 4 patients, and a mild-moderate depression (BDI score between 10 and 18 points) in 1 patient.

### 3.4. Assessment of the Coping Strategies

The coping strategies of patients with relevant depressive disorder and of those without any psychiatric symptoms were assessed through the Brief-COPE.

We found significant differences in coping styles adopted by these groups. In particular, patients with depressive symptoms showed lower values of “positive reframing” (*p* = 0.017) that refers to the items “I try to see it in a different light, to make it seem more positive” and “I look for something good in what is happening” and lower values in “planning” (*p* = 0.046) that corresponds to the items “I try to come up with a strategy about what to do” and “I think hard about what steps to take.” Patients with depressive comorbidity showed conversely higher points in “use of instrumental social support” (*p* < 0.001) corresponding to the items “I talk to someone to find out more about the situation” and “I try to get advice from someone about what to do,” in the “denial” scale (*p* = 0.001) that refers to the items “I say to myself this isn't real” and “I pretend that it hasn't really happened,” and in the one of “use of emotional social support” (*p* = 0.003) that includes the items “I try to get emotional support from friends or relatives” and “I get sympathy and understanding from someone” (Table 2).

## 4. Discussion

Our study confirmed that psychiatric comorbidity is a frequent condition in CD patients even in those in clinical remission, confirming previous data [5] and that depressed CD patients have altered coping strategies.

It is well known that CD is associated with higher rates of anxiety and mood disturbances compared to the general population and the European Crohn's and Colitis Organisation has underlined the importance to identify the patients that need a psychiatric intervention, recommending an interdisciplinary approach between gastroenterologist and psychiatrist [34, 35]. In particular, we observed that 16.8% of CD patients in clinical remission showed depressive symptoms and nearly half of them (43.8%) corresponding to the 7.3% of the overall study population had a definite depression. However, considering that these data come from a series of CD patients in clinical remission and that anxiety and depression are far more frequent patients with active CD [6, 36], our data likely underestimate the real prevalence of psychiatric disorders in CD and therefore their impact on coping strategies in CD patients.

Regarding the analysis of coping strategies in CD patients we showed that patients with depression have significantly lower levels of “positive reframing” and “planning” and significantly higher levels of “denial” compared to CD patients without psychiatric comorbidity. This means that CD patients, before a stressing situation, tend to become demoralized, are not able to implement functional strategies to manage at best the stress, and, actually, prefer denying problems and avoiding them. This attitude reflects on patients' behaviour, resulting in a reduced compliance to treatments and medical prescriptions, which is reported in 20% of patients with CD [37–40].

Patients with depression show significantly higher levels of “use of emotional support” and “use of instrumental support”; this means a deep dependence on the others, requirement to find outside the resources to cope with stressing situations in terms of both sympathy or comfort and advices. Therefore the characteristics of passivity in their way to cope with the stress are evident. This data is in keeping with other preliminary studies that have shown a correlation between passive coping and depression in patients with IBD and CD in particular [41, 42].

Other studies highlighted the relation between coping strategies and depression and suggested that a dysfunctional coping style reduces the tolerance of physical symptoms and worsens patients' quality of life and depressive disorders. On the other hand, the typical manifestations of the depressive disorder, like loss of interests, self-devaluation, and difficulty in taking decisions, contribute to the patients use of passive coping strategies such as avoidance, catastrophization, and churning [15, 19, 21].

Crohn's disease is characterized by alternate periods of wellbeing and symptoms that can change look and social and working functioning and usually cause significant recurring stressors in patients. Considering that depression is frequent in CD patients [2–4] most of them likely have a higher probability to cope with stressing situations such as steroidal and immunosuppressive therapies, hospitalizations, or surgical operations. Therefore, interventions focused on coping strategies should be considered as integrated treatment for CD patients in order to improve their psychological wellbeing. Accepting the disease as part of one's life and trying to get from the experience constructive aspects could result in being essential to set new objectives and find motivational resources to pursue them [43, 44]. On this regard, cognitive behavioural therapy could be a useful resource in CD patients [45, 46]. Interventions of coping skills therapy have been already shown to be useful in improving capacity of problem-solving and patients' psychological wellbeing [47]. However, recent results investigating the effect of cognitive behavioural therapy or other supportive therapies in patients with IBD and depression suggested improvements of psychological distress, global functioning, and quality of life [48, 49]. Besides these treatments, presently there are few data on the efficacy of antidepressants and anxiolytics in CD, although recent studies showed that antidepressants are associated with a favourable outcome of the disease in IBD patients [50, 51]_._ Therefore, it is considered now important to approach CD patients with depressive disorders intervening by means of a pharmacologic and psychotherapeutical treatments in order to improve compliance to the treatment, clinical features of CD, and overall the patients' quality of life.

Limitations of this study are the lack of data regarding the possible risk factors for depression and the impact of the socioeconomic level of patients on coping strategies (e.g., the family income and the schooling level), and in turn the effect of these on compliance to the treatment and overall quality of life. These can be matter of future studies. Furthermore, in order to answer the Brief-COPE questionnaire the patients were requested to generalize their way to react to stressing situations while the coping strategies can be partially changed according to the event to cope with.

Finally, the major limitation in this study is that the analysis was based on the comparison with only seven patients with confirmed depression, a limited numerosity due to strict selection of patients with confirmed depression. In conclusion this study showed that CD patients in clinical remission may have significant rate of anxiety and depressive symptoms. The latter may be untreated and associated with altered coping strategies. This potentially means that patients may not be able to implement functional strategies to manage at best stress related with their disease. This confirms again that CD patients may need an interdisciplinary approach between gastroenterologist and psychiatrist.

## Figures and Tables

**Table 1:** Demographic and clinical features of patients included in the study.

Sex male, *n* (%)	57 (60)
Age (years), mean (SD)	45 (14)
Age at diagnoses of CD (years), mean (SD)	28 (10)
Disease length (years), mean (SD)	20 (12)
Family history of IBD, *n* (%)	16 (16.8)
Treatment, *n* (%)	
Immunosuppressive treatment	36 (37.9)
Treatment with anti-TNF-alpha	13 (13.7)
Mesalazine	24 (25.3)
No therapy (clinical observation)	22 (23)
Crohn's disease's localization *n* (%)	
Ileum	42 (44.2)
Colon	13 (13.7)
Ileocolonic	40 (42.1)
Isolated upper disease	3 (3.2)
Crohn's disease's behaviour *n* (%)	
Nonstricturing, nonpenetrating	28 (29.5)
Stricturing	45 (47.4)
Penetrating	22 (23.1)
History of perianal disease	10 (10.5)
Surgery, *n* (%)	
No surgery	35 (36.8)
Conservative surgery	10 (10.5)
Resective surgery	50 (52.6)
History of surgery for perianal disease	10 (10.5)

**Table 2:** Coping strategies in CD patients with depression and those without psychiatric disorders expressed as median of the scores of each item considered in the brief COPE.

	Pts. with depression	Pts. without psychiatric disorders	*p* value
	(*n* = 7)	(*n* = 58)	
	Median (Q_25_–Q_75_)	Median (Q_25_–Q_75_)	
Positive reframing	4 (2.25–5.75)	6 (5–7)	**0,017**
Self-distraction	6 (4–6)	5 (4–7)	0,732
Venting	6 (4.25–7.75)	5 (4-5)	0,085
Use of instrumental support	7 (6–8)	5 (4–6)	**<0,001**
Active coping	6 (6–8)	7 (5–8)	0,793
Denial	5 (4–6)	2 (2–4)	**0,001**
Religion	6 (3.5–6.75)	4 (2–6)	0,223
Humour	3 (2.25–4.75)	4 (3–5)	0,149
Behavioural disengagement	3 (2–4)	2 (2–4)	0,713
Use of emotional support	7 (6-7)	4 (3–5)	**0,003**
Substance use	2 (2-2)	2 (2-2)	0,345
Acceptance	7 (6.25–8)	8 (6–8)	0,891
Planning	5 (3.25–6)	6 (5–8)	**0,046**
Self-blame	3 (3–5)	4 (4-5)	0,286
